# Implementing RSA for Wireless Sensor Nodes

**DOI:** 10.3390/s19132864

**Published:** 2019-06-27

**Authors:** Utku Gulen, Abdelrahman Alkhodary, Selcuk Baktir

**Affiliations:** 1 College of Engineering and Technology, American University of the Middle East, Egaila 15453, Kuwait; 2 Computer Engineering, Faculty of Engineering and Natural Sciences, Bahcesehir University, Istanbul 34353, Turkey

**Keywords:** wireless sensor networks, RSA, subtractive Karatsuba-Ofman, montgomery multiplication, Chinese remainder theorem

## Abstract

As wireless sensor networks (WSNs) become more widespread, potential attacks against them also increase and applying cryptography becomes inevitable to make secure WSN nodes. WSN nodes typically contain only a constrained microcontroller, such as MSP430, Atmega, etc., and running public key cryptography on these constrained devices is considered a challenge. Since WSN nodes are spread around in the field, the distribution of the shared private key, which is used in a symmetric key cryptographic algorithm for securing communications, is a problem. Thus, it is necessary to use public key cryptography to effectively solve the key distribution problem. The RSA cryptosystem, which requires at least a 1024-bit key, is the most widely used public key cryptographic algorithm. However, its large key size is considered a drawback for resource constrained microcontrollers. On the other hand, RSA allows for extremely fast digital signature generation which may make it desirable in applications where messages transmitted by sensor nodes need to be authenticated. Furthermore, for compatibility with an existing communication infrastructure, it may be desirable to adopt RSA in a WSN setting. With this work, we show that, in spite of its long key size, RSA is applicable for wireless sensor networks when optimized arithmetic, low-level coding and some acceleration algorithms are used. We pick three versions of the MSP430 microcontroller, which is used widely on wireless sensor network nodes, and implement 1024-bit RSA on them. Our implementation achieves 1024-bit RSA encryption and decryption operations on MSP430 in only 0.047 s and 1.14 s, respectively. In order to achieve these timings, we utilize several acceleration techniques, such as the subtractive Karatsuba-Ofman, Montgomery multiplication, operand scanning, Chinese remainder theorem and sliding window method. To the best of our knowledge, our timings for 1024-bit RSA encryption and decryption operations are the fastest reported timings in the literature for the MSP430 microcontroller.

## 1. Introduction

A wireless sensor network (WSN) consists of a large number of sensor nodes which are usually controlled by a constrained microcontroller, e.g., 16-bit MSP430, 8-bit Atmega, etc. Applications of WSNs vary widely from environmental practices to health, and from industrial control applications to military purposes [1,2,3,4]. WSNs also find applications in areas such as multimedia networks [5] and smart grid networks [6]. WSN applications in diverse fields rely on underlying algorithms, protocols and standards [7]. Moreover, many WSN applications need data security and confidentiality since sensitive information is stored, processed or transferred by sensor nodes [8,9]. As the number and diversity of WSN applications grow, so do the variety of their security and privacy issues [10,11,12]. Therefore, it is a necessity to implement cryptographic schemes efficiently on constrained microcontrollers used in sensor nodes [13,14,15]. Microcontrollers on WSN nodes are typically limited by their memory capacity and CPU speed. Besides, energy efficiency is another important constraint since WSN nodes are either battery powered or they environmentally harvest their energy [16,17,18,19,20]. All these constraints make implementing cryptography, more particularly complex public key cryptographic operations, on WSN nodes a major challenge.

For providing security to WSN nodes, symmetric key cryptography may seem to be a feasible solution at first glance. However, symmetric key cryptography alone would not be a remedy for providing security to WSN nodes. WSN nodes are typically placed far apart from each other and the distribution of shared symmetric keys is a challenge. For distributing the shared symmetric keys efficiently, public key cryptography (PKC) is needed [21,22,23,24]. Furthermore, PKC makes it possible to electronically sign digital messages [25]. In WSNs, malicious nodes can inject false or counterfeit messages into the network. Hence, implementation of an authentication mechanism would be necessary to prevent these messages. Digital signatures, provided by PKC, can be used to authenticate messages exchanged between sensor nodes. Although the computational complexity of PKC is considered a drawback for WSN nodes with constrained microcontrollers, previous works show that PKC can be a viable option [26,27,28,29,30,31]. The two most popular PKC schemes are elliptic curve cryptography (ECC) and the Rivest Shamir Addleman (RSA) cryptosystem [25,32,33]. Many of the previous PKC implementations for WSNs in the literature prefer ECC due to its shorter key size [34,35,36]. ECC requires at least a 160-bit key to be considered secure. In RSA, on the other hand, the same level of security can be achieved with a 1024-bit key. However, despite its larger key size, RSA is more widely used particularly by general purpose computers in Internet applications. Therefore, if a system provider plans to develop a new system for WSNs which should be compatible with an existing communication infrastructure that uses RSA, it would be desirable to adopt RSA. Another advantage of RSA is that by choosing a small public key, signature verification can be achieved faster and may consume less energy compared to other public key algorithms including ECC [37]. Hence, it may be desirable to use RSA for WSN applications where extremely fast authentication is required for messages transmitted by sensor nodes. There are existing 1024-bit RSA implementations similar to our work [38,39,40,41]. Our aim in this work is to implement 1024-bit RSA for WSNs more efficiently than existing works in the literature.

**Main Contributions:**

1. We use the subtractive Karatsuba-Ofman, Montgomery multiplication, CRT and operand scanning algorithms together for the first time in the literature, and implement RSA using these.
2. Our RSA encryption and decryption implementations on the MSP430 microcontroller have the fastest timings in the literature.
3. We show that faster RSA timings are feasible on WSN nodes and the RSA cryptosystem may be a preferable PKC option for WSNs.

## 2. Background

### 2.1. The RSA Cryptosystem

The RSA public key cryptosystem [25], introduced by Rivest, Shamir and Adleman in 1978, is the first ever and still most widely used general purpose public key cryptographic algorithm. In RSA, encryption and decryption are described by the below equations where *x* is the plaintext, *y* is the ciphertext, *e* is the public encryption key, *N* is part of the public key and *d* is the private (decryption) key.

(1) $$\begin{array}{c} RSA\;Encryption:\;\;y=x^{e}\;\mathrm{mod}\;N \end{array}$$

(2) $$\begin{array}{c} RSA\;Decryption:\;\;x=y^{d}\;\mathrm{mod}\;N \end{array}$$

The modulus *N*, which is used for performing modular arithmetic, is the product of two large primes, denoted with *p* and *q* [25]. Furthermore, there is the following relationship between *e* and *d*:

(3) $$\begin{array}{c} e=d^{-1}\;\mathrm{mod}\;\phi \left(N\right), \end{array}$$

where ϕ(N), the Euler’s Phi function, has the following value:

(4) $$\begin{array}{c} \phi (N)=(p-1)\times (q-1)\;. \end{array}$$

If the public key *N* can be factorized into *p* and *q*, it would be possible to compute ϕ(N)=(p−1)×(q−1) and obtain the secret decryption key *d* by computing $e^{-1}\;\mathrm{mod}\;\phi \left(N\right)$. Hence, the security of RSA relies on the difficulty of factorizing the modulus *N*. If *N* is large enough, it cannot be factorized to obtain *d* and thus RSA cannot be broken. For this reason, we implement RSA with 1024-bit keys where all arithmetic is performed over 1024-bits numbers. We utilize several algorithms, described in the following, to speed up large integer arithmetic with 1024-bits numbers.

### 2.2. Chinese Remainder Theorem

Applying the Chinese remainder theorem (CRT) is a common method used to speed up RSA decryption [42]. Using the CRT, for any integer value *y*, ymodN can be uniquely represented as $\left(y_{p},y_{q}\right)$ where $y_{p}$ and $y_{q}$ are the residues of *y* modulo the relatively prime numbers *p* and *q*, respectively. Note that the CRT representation can be used in RSA decryption due to the fact that the RSA modulus *N* is the product of two prime numbers. The normal integer representation of ymodN can be recovered from its CRT representation using the formula

(5) $$\begin{array}{c} y=\left(q\times c_{p}\right)\times y_{p}+\left(p\times c_{q}\right)\times y_{q}\;\mathrm{mod}\;N, \end{array}$$

where

(6) $$\begin{array}{cc} c_{p} & =q^{-1}\;\mathrm{mod}\;p\;\;\;\mathrm{and}\;\;\;c_{q}=p^{-1}\;\mathrm{mod}\;q\;. \end{array}$$

Modular multiplication, as it takes place in RSA decryption, can be done in the CRT representation using the moduli *p* and *q*, instead of the usual RSA modulus *N*. Furthermore, repeated multiplications modulo *N*, as it takes place in RSA decryption, can be performed in the CRT representation and the final result can be converted back to the normal integer representation. Hence, the RSA decryption operation, i.e., the computation of $x=y^{d}\;\mathrm{mod}\;N$ can be achieved using the CRT as

$$\begin{array}{cc} x_{p} & =y_{p}^{d_{p}}\;\mathrm{mod}\;p, \end{array}$$

(7) $$\begin{array}{cc} x_{q} & =y_{q}^{d_{q}}\;\mathrm{mod}\;q, \end{array}$$

(8) $$\begin{array}{cc} x & =\left(q\times c_{p}\right)\times x_{p}+\left(p\times c_{q}\right)\times x_{q}\;\mathrm{mod}\;N, \end{array}$$

where $d_{p}=d\;\mathrm{mod}\;\left(p-1\right)$ and q=dmod(q−1). Note that the computations of $x_{p}=y_{p}^{d}$ and $x_{q}=y_{q}^{d}$ are simplified as $y_{p}^{d_{p}}$ and $y_{q}^{d_{q}}$, respectively, by taking advantage of the Euler’s theorem which states that $y_{p}^{d}\equiv y_{p}^{d\mathrm{mod}\;\phi (p)}\left(\mathrm{mod}\;p\right)$ and $y_{q}^{d}\equiv y_{q}^{d\mathrm{mod}\;\phi (q)}\left(\mathrm{mod}\;q\right)$.

In the above RSA decryption operation using the CRT, the values $d_{p}$, $d_{q}$, $q\times c_{p}$, $p\times c_{q}$, $c_{p}$ and $c_{q}$ can be precomputed. Furthermore, in (9), note that the bit-lengths of the operands that are exponentiated, as well as the exponents, are half their sizes in the normal RSA decryption without the CRT. Therefore, using the CRT reduces the overall timing of RSA decryption dramatically by a factor of up to four.

### 2.3. Sliding Window Method

The exponentiation operation for RSA decryption can be achieved by using the basic binary scan method [42]. This method scans the bits of the exponent one bit at a time starting with the most significant non-zero bit. For the most significant non-zero bit, the intermediary result is initially set to the value of the base. Then, for each new bit scanned, the intermediary result is updated with its square. If the newly scanned bit is 1, then the intermediary result is further updated by multiplying it with the base value. All arithmetic operations, i.e., multiply and square, are performed in modulo the RSA modulus *N*. In this method, the number of square operations conducted is equal to one less than the number of bits in the exponent. The number of multiply operations is equal to one less than the Hamming weight of the exponent, which, on average, is half the bit-length of the exponent minus 1. Hence, for a *t*-bit RSA decryption operation, the basic binary scan method performs t−1 modular squarings and on average (t−1)/2 modular multiplications.

The sliding window method for exponentiation improves upon the binary scan method by reducing the number of required modular multiplications. In Algorithm 1, the sliding window method is given for a 4-bit window. The base and the exponent are represented with *b* and *e*, respectively.

**Algorithm 1:** 4-bit Sliding Window Method

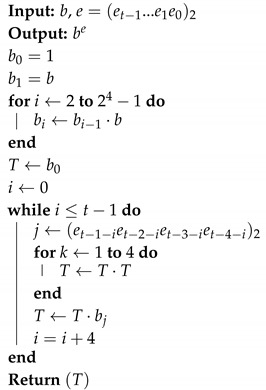



### 2.4. Montgomery Multiplication

Since arithmetic is performed in modulo *N* for RSA encryption and decryption, the result of every multiplication operation needs to be reduced modulo *N*. The Montgomery multiplication algorithm [43], given in Algorithm 2, can be used to achieve multiplication modulo *N* efficiently. For multiplying the two integers *P* and *Q* with the algorithm, they should be converted into their Montgomery forms as $\bar{P}$ and $\bar{Q}$, where $\bar{P}=P\times r\;\mathrm{mod}\;N$ and $\bar{Q}=Q\times r\;\mathrm{mod}\;N$. Here the constant *r* can be picked to be any integer that is greater than and relatively prime with *N*. For purposes of efficiency, *r* can be chosen to be the smallest power of 2 that is greater than *N*. Montgomery multiplication of $\bar{P}$ and $\bar{Q}$ produces $\bar{P}\times \bar{Q}\times r^{-1}\;\mathrm{mod}\;N$ which is the Montgomery form $\bar{R}$ of the product R=P×QmodN. Thus, Montgomery multiplication preserves the Montgomery form and repeated multiplications, such as the multiplications that make up an exponentiation, can be performed using Montgomery multiplication.

The arithmetic operations performed for achieving Montgomery multiplication are integer multiplication, addition and subtraction. Subtraction is computed only when the intermediary result *T* is larger than or equal to *N*. The cost of the division operation by $2^{n}$, on line 3 of Algorithm 2, can be ignored since the first *n* bits of *T* are zeroes and the higher bits readily give the result.

**Algorithm 2:** Montgomery Multiplication**Input**: $\bar{P}=P\times r\;\;\mathrm{mod}\;N$ and $\bar{Q}=Q\times r\;\;\mathrm{mod}\;N$
**where**
$r=2^{n}$ and $n=\lceil \log_{2}N\rceil$ **Output**: $\bar{R}=P\times Q\times r\;\mathrm{mod}\;N$ $S\leftarrow \bar{P}\times \bar{Q}$ $W\leftarrow S\times N^{\prime }\;\mathrm{mod}\;r$
**where**
$N^{\prime }=N^{-1}\;\;\mathrm{mod}\;r$ T←(S+W×N)/r **if**
T≥N
**then**(( |   T=T−N **end** ( R←T **Return**
$\left(\bar{R}\right)$

### 2.5. Subtractive Karatsuba-Ofman

**Figure 1:**
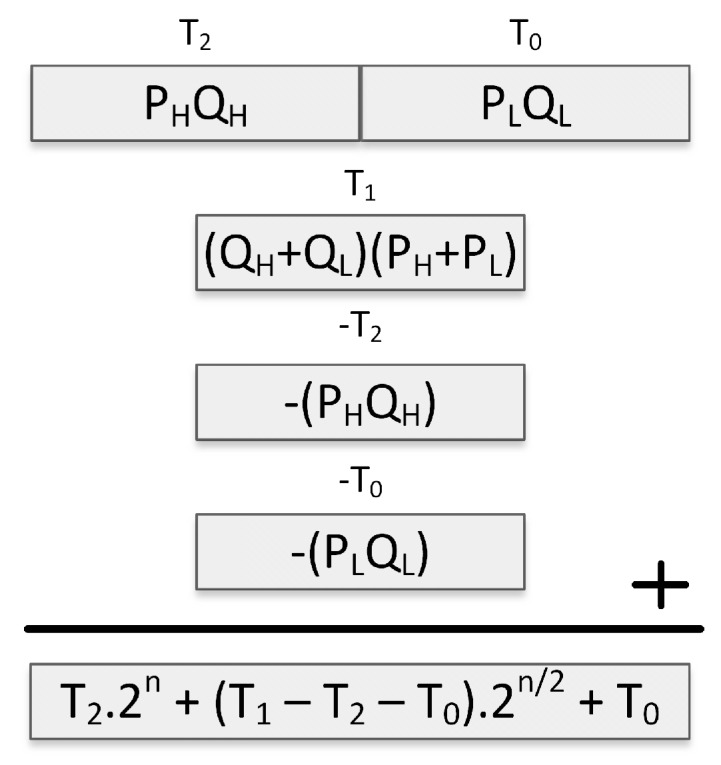
Karatsuba-Ofman method for computing P×Q.

Karatsuba-Ofman method is a technique to accelerate integer multiplication [44]. It achieves multiplication of two integers by performing multiplications and additions/subtractions of smaller integers. Figure 1 shows the Karatsuba-Ofman method applied for the multiplication of the two integers *P* and *Q*. In the method, each operand is first fragmented into two halves containing the higher and lower ordered bits. The higher and lower ordered halves of *P* and *Q* are represented as $P_{H}$, $P_{L}$, $Q_{H}$ and $Q_{L}$. Three partial products, i.e., $T_{0}$, $T_{1}$ and $T_{2}$, are computed by adding/multiplying the operand halves. Finally, with some additions and subtractions on the partial products, the result R=P×Q is obtained, as shown in Figure 1.

**Figure 2:**
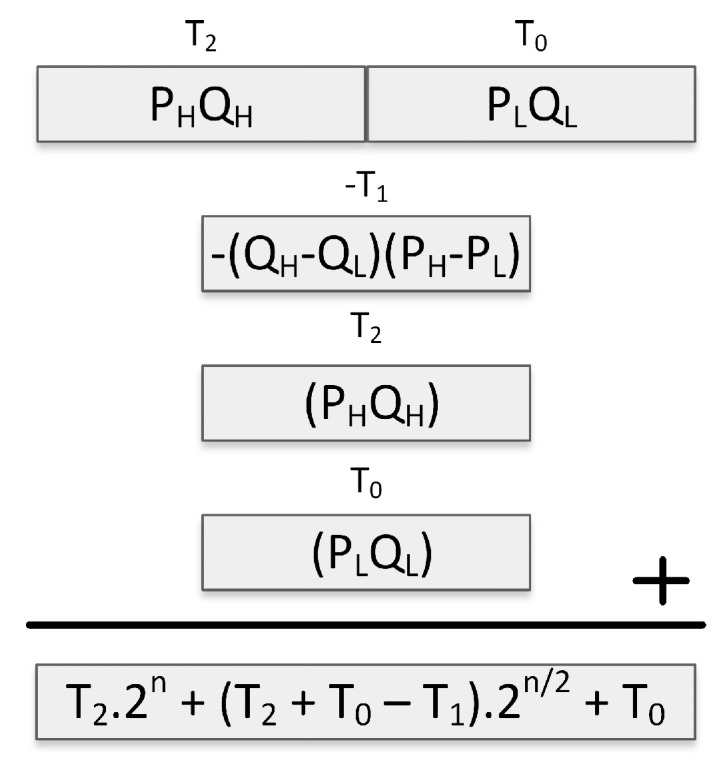
Subtractive Karatsuba-Ofman for computing P×Q.

Since the algorithm adds higher and lower ordered halves of the operands and multiplies them to compute $T_{1}$, the size of this multiplication may alter depending on the carry bits generated by the additions. For this reason, integer multiplication may cost slightly more in this step. Subtractive Karatsuba-Ofman, which is an improved version of Karatsuba-Ofman, was proposed to mitigate this problem [45,46]. The subtractive Karatsuba-Ofman technique, described in Figure 2, eliminates the carry bits that are generated by the addition operations.

Subtractive Karatsuba-Ofman ensures that the multiplication operation that is performed to generate $T_{1}$ is fixed since subtractions are performed instead of additions, which do not generate carry bits. However, a final negation operation is required on $T_{1}$ if it ends up being negative. Negation is straightforward and can be computed by only performing XOR operations with the value 0× FFFF on all the 16-bit words of $T_{1}$ on a 16-bit processor (such as MSP430). This XOR operation can be performed regardless of the sign of $T_{1}$ by storing a flag word which becomes 0× FFFF or 0×0000 according to the sign of $T_{1}$ [45]. If $T_{1}$’s sign is positive, the XOR operation with 0×0000 does not change the result, however, it serves the purpose of keeping the execution time fixed.

## 3. Our RSA Implementation on MSP430

Texas Instruments MSP430 microcontroller is deployed on WSN nodes such as TMote Sky, TelosB, AS-XM1000, etc. It is a low power and low cost microcontroller which has RISC architecture with 12 general purpose registers. In this work, we use three versions of MSP430, namely MSP430F1611 [47], MSP4302618 [48] and MSP430F5529 [49], as our target platform for implementing 1024-bit RSA and IAR Embedded Workbench as our development environment [50]. We use C for high-level coding and assembly instructions for core arithmetic operations. All three versions of MSP430 that we use support the common MSP430 instruction set. However, MSP4302618 and MSP430F5529 have additional instructions known as MSP430X instructions which allow using 20-bit address space for some instructions. Nevertheless, we use only the basic MSP430 instruction set for our assembly subroutines, so that we can run the same assembly code on all three microcontrollers. We would like to note that the MSP430 versions which support MSP430X run the same code slightly faster since they execute the memory write instruction in 3 clock cycles while the other MSP430 versions execute the same instruction in 4 clock cycles. The clock speeds for MSP430F1611, MSP430F2618 and MSP430F5529 are 8 MHz, 16 MHz and 25 MHz, and their memory capacities are 48 kB, 116 kB and 128 kB, respectively. MSP430F5529 has an integrated 32×32-bit hardware multiplier while the other two MSP430 versions have only a 16×16-bit hardware multiplier. Therefore, for MSP430F5529, we modify our implementation to utilize the 32×32-bit hardware multiplier.

In our 1024-bit RSA implementation, we use several optimizations and algorithms, some of which are described in Section 2, to accelerate the timings for encryption and decryption operations. We use the CRT and the sliding window method [42] to speed up RSA decryption and a small exponent, i.e., $e=2^{16}+1$, to speed up RSA encryption [42]. Furthermore, we use Montgomery multiplication [43,51] with optimized integer multiplication to speed up both the encryption and decryption operations. We use the subtractive Karatsuba-Ofman method [44,45] recursively to facilitate fast integer multiplication which helps accelerate the overall Montgomery multiplication operation. Furthermore, we optimize the subtractive Karatsuba-Ofman method for speeding up integer squaring. For the multiplications and squarings at the base of recursion in our subtractive Karatsuba-Ofman implementations, we use the operand scanning method. Moreover, we optimize the operand scanning method for speeding up integer squaring.

### 3.1. Exponentiation Using Sliding Window Method, CRT and Montgomery Multiplication

For the exponentiation operation in RSA encryption, we use the small exponent $e=2^{16}+1$. Thus, only 16 modular squarings and 1 modular multiplication are needed for RSA encryption. However, RSA decryption needs to be computed using a random exponent that is 1024-bits in length, which means a large number of modular multiplications/squarings should be computed.

For the exponentiation operation in RSA decryption, we use the sliding window method given in Section 2.3 and the CRT given in Section 2.2. Using the CRT, a 1024-bit exponentiation modulo N=p×q, as it takes place in RSA decryption, can be achieved by performing only two 512-bit remainder computations, two 512-bit modular exponentiations, two 1024×512-bit multiplications, one 1536-bit addition modulo *N*. Hence, the cost of 1024-bit modular exponentiation, for RSA decryption, using the CRT, is roughly two 512-bit modular exponentiations. Note that for computing a *t*-bit modular exponentiation, around 5t/4 modular multiplications/squarings are performed in the 4-bit sliding window method. Our operand size for exponentiation is 512 bits for RSA decryption (due to the use of the CRT). Using the 4-bit sliding window method, a 512-bit modular exponentiation operation can be achieved by performing 7 modular squarings and 7 modular multiplications to fill up the 4-bit lookup table, a total number of 508 modular squarings for the 507th down to 0th scanned bits of the exponent and a total number of 127 modular multiplications for the 4-bit windows of the exponent that are scanned. Hence, a total number of 515 512-bit modular squarings and 134 512-bit modular multiplications are performed to achieve a 512-bit exponentiation. For RSA decryption using the CRT, two 512-bit modular exponentiations are performed, hence the cost of decryption is roughly 1030 512-bit modular squarings and 268 512-bit modular multiplications.

In addition to its timing performance, another advantage of the sliding window method over the binary scan method is that it obscures the exponent’s bits against simple power analysis [52]. The sliding window method performs the same set of operations for every scanned window, whereas the binary scan method performs one squaring for every scanned bit and an additional multiplication only when the scanned bit is 1. Therefore, in the binary scan method the bits of the exponent can be predicted easily by merely observing the consumed power during exponentiation.

In our sliding window method implementation, we realize modular multiplication by using Montgomery multiplication (described in Section 2.4) [43]. For modular squaring, we use an optimized version of Montgomery multiplication which we call as Montgomery squaring. In order to speed up the integer multiplications performed in Montgomery multiplication and squaring, we use subtractive Karatsuba-Ofman.

### 3.2. Fully-Recursive Subtractive Karatsuba-Ofman for Multiplication and Squaring

We realize the 1024-bit integer multiplication/squaring operation (for RSA encryption) and the 512-bit integer multiplication/squaring operation (for RSA decryption) by applying the subtractive Karatsuba-Ofman method recursively. We use 4 and 3 levels of recursion for 1024-bit and 512-bit integer multiplications/squarings, respectively. We observe that, on our target microcontroller which has a 16×16-bit hardware multiplier, there is no further speed up with more levels of recursion in subtractive Karatsuba-Ofman after we reach the 64-bit inner multiplications/squarings. For the operand size of 64 bits, we observe that operand scanning has better timing performance than subtractive Karatsuba-Ofman. Therefore, we realize these 64-bit multiplications/squarings, at the base case of recursion, by using the operand scanning method.

For our other target microcontroller with a 32×32-bit hardware multiplier, we observe that operand scanning has better timing performance than subtractive Karatsuba-Ofman for operand sizes less than or equal to 128 bits. Therefore, on this microcontroller, we achieve 1024-bit integer multiplication/squaring operations (for RSA encryption) and the 512-bit integer multiplication/squaring operations (for RSA decryption) by applying the subtractive Karatsuba-Ofman method for 3 and 2 levels of recursion, respectively. At the base case of recursion, where the operand size is 128 bits, we realize the integer multiplications/squarings using operand scanning.

**Figure 3:**
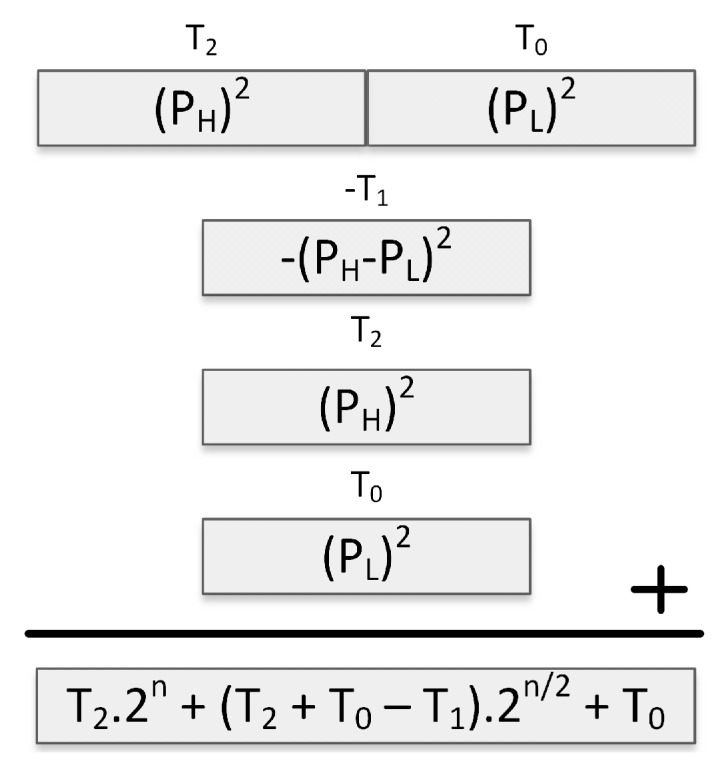
Subtractive Karatsuba-Ofman for computing $P^{2}$.

As shown in Figure 3, we optimize the subtractive Karatsuba-Ofman method for integer squarings by omitting some computations in the original subtractive Karatsuba-Ofman method. For instance, $T_{1}$ will always have a non-negative value since it is the result of a squaring operation, i.e., ${\left(P_{H}-P_{L}\right)}^{2}$. Therefore, unlike in multiplication, the sign of $T_{1}$ is known in advance, and there is no need to perform a negation before subtracting $T_{1}$ from $T_{0}+T_{2}$.

### 3.3. Multiplication and Squaring with Operand Scanning

We use the operand scanning method to perform the 64-bit multiplications/squarings at the base of our 4 and 3 level recursive subtractive Karatsuba-Ofman implementations for 1024-bit and 512-bit integer multiplication/squaring operations, respectively. Operand scanning, also known as schoolbook multiplication, is the classical method for multiprecision integer multiplication. On the 16-bit MSP430, we can achieve 64-bit multiplication with operand scanning by storing the two 64-bit operands in the 8 general purpose registers. The other 4 general purpose registers are used for the execution of operand scanning and for storing half of the result. The second half of the result is written to the operand registers as they become available later during the processing. Thus, 64-bit multiplication can be handled by using only the general purpose registers and without the use of memory which would have otherwise slowed down the multiplication operation. If the subtractive Karatsuba-Ofman method was used for this 64-bit multiplication operation, the partial products would have to be stored in the memory and also read from the memory, resulting in increased time cost. Our MSP430 microcontroller has a memory mapped hardware multiplier. This hardware multiplier is accessed through special function registers and the timing cost of multiplication using the hardware multiplier is only the time it takes to write the operands to the input registers and read the result from the output registers.

**Figure 4:**
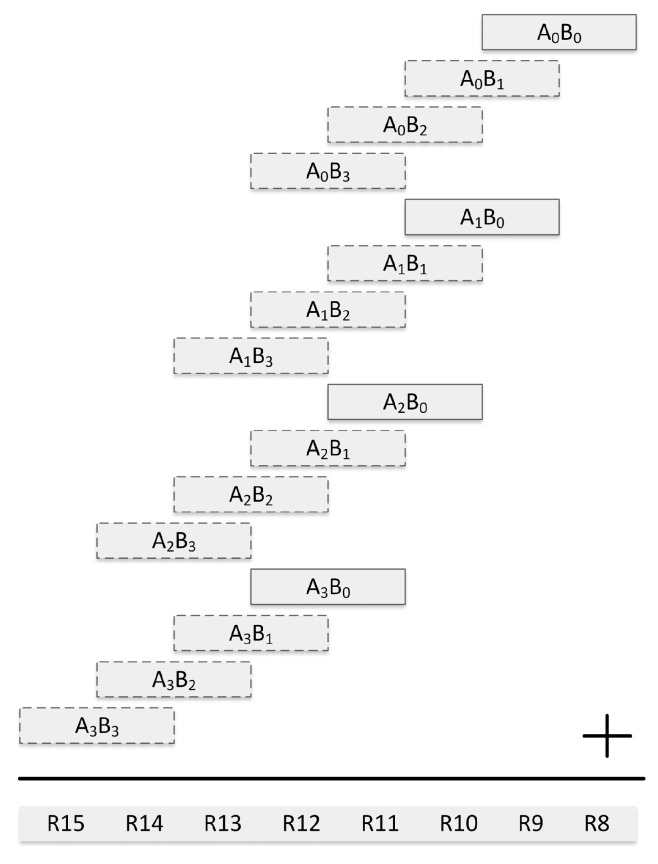
Operand scanning for multiplying two 64-bit operands on a 16-bit processor.

Figure 4 gives the order of word multiplications in our operand scanning implementation on the 16-bit MSP430 microcontroller. The 64-bit operands *A* and *B* are represented in four words as [$A_{3},A_{2},A_{1},A_{0}$] and [$B_{3},B_{2},B_{1},B_{0}$], respectively. The method computes word multiplications from top to down as described in Figure 4. By doing this, the words $A_{3}$, $A_{2}$, $A_{1}$ and $A_{0}$ are written to the hardware multiplier register only once. As seen in Figure 4, $A_{0}$ is the first operand for the first four multiplications and it is written to the multiplier register only once. Whereas, four write operations are performed to write the words of the second operand, i.e., $B_{3}$, $B_{2}$, $B_{1}$ and $B_{0}$, to the second input register of the hardware multiplier. Similarly, register write operations are saved also for the multiplications with $A_{1}$, $A_{2}$ and $A_{3}$ in following 12 word multiplications. The word multiplications where a register write operation is saved are indicated in Figure 4 with rectangles surrounded by a dashed border line. The 128-bit final result is stored in the registers R15 down to R8.

**Figure 5:**
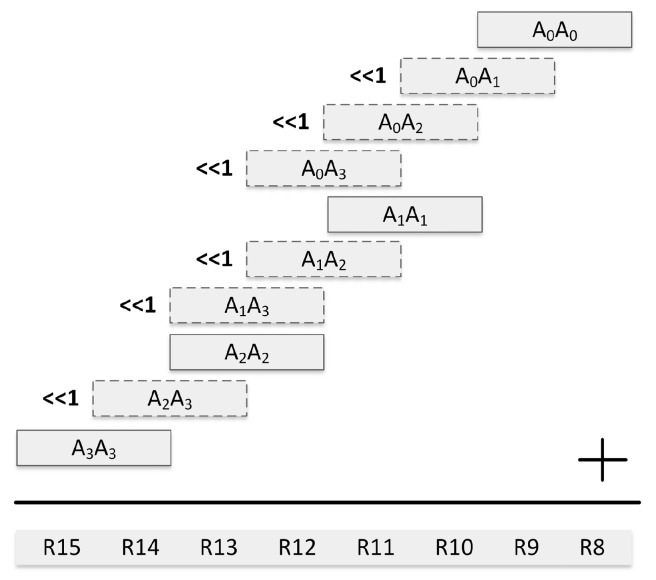
Operand scanning for squaring a 64-bit operand on a 16-bit processor.

We optimize the operand scanning method for the 64-bit squaring operation as shown in Figure 5. Note that, similar to operand scanning multiplication, register write savings in operand scanning squaring are indicated with dashed border lines in Figure 5. In 64-bit operand scanning squaring, the number of word multiplications can be reduced to 10 by performing a 1-bit shift operation on the results of some word multiplications instead of recomputing and adding the same word products.

For our RSA implementation on MSP430F5529, which has a 32×32-bit hardware multiplier, we perform operand scanning multiplication/squaring similarly but for 128-bit operands since each word multiplication here is a 32×32-bit multiplication. For this scenario, we achieve 1024-bit and 512-bit integer multiplication/squaring operations by applying the subtractive Karatsuba-Ofman method recursively for 3 and 2 levels of recursion, respectively, and we achieve the 128-bit inner multiplications/squarings using operand scanning. Similar to the case with the 16×16-bit hardware multiplier and 64-bit operand size, we apply operand scanning on operands with four words, but this time the operands are 128 bits and the words of the operands are 32 bits in length. Here, unlike in the case with the 64-bit operand size, there are not enough registers on the microcontroller to store the two 128-bit operands. Therefore, for 128-bit operand scanning, we simply read the operands from the memory, calculate the necessary word multiplications using the 32×32-bit hardware multiplier and then store the partial results in the registers. Since the final product is 256-bit long, we write the lower half of the result into the memory when it is computed. This allows us to free registers to be used for storing the following partial results. Thus, except for not storing all operands in registers, we realize the same computations as for 64-bit operand scanning on MSP430 with 16×16-bit hardware multiplier.

**Figure 6:**
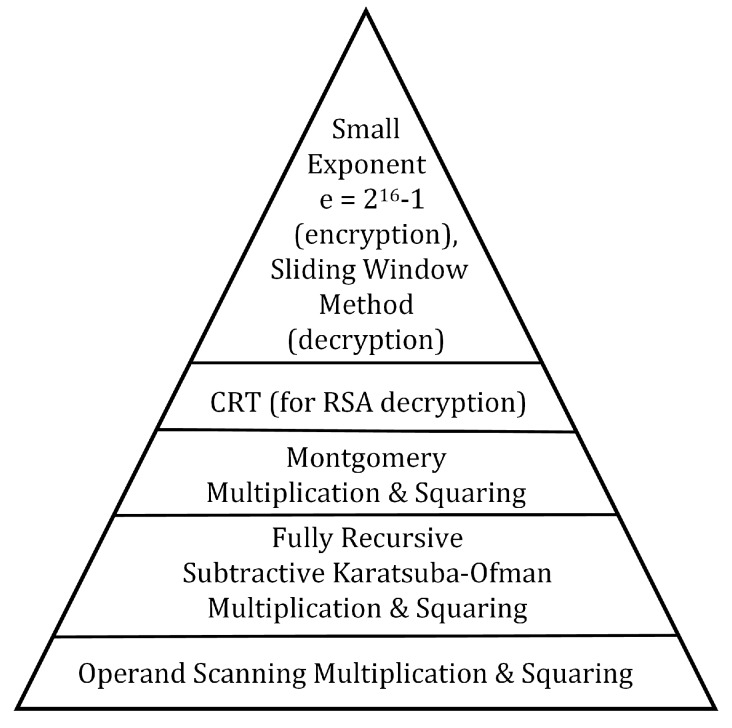
A bottom-up schema of the techniques used in the proposed RSA implementation.

To summarize our RSA implementation, in Figure 6, we visualize the various steps of our implementation from the bottom up. The underlying core arithmetic operations, which make the biggest difference in the performance of our RSA encryption/decryption implementations, are the operand scanning multiplication/squaring and fully recursive subtractive Karatsuba-Ofman algorithms. We achieve these core arithmetic operations, as well as addition and subtraction operations, in the assembly language, and realize Montgomery modular multiplication/squaring on top of them. We achieve RSA encryption using a small public exponent which requires performing a small number of Montgomery multiplication/squaring operations. For RSA decryption, we utilize the CRT while performing the required Montgomery multiplication/squaring operations. Furthermore, we use the sliding window method to reduce the number of required Montgomery multiplications for RSA decryption.

## 4. Implementation Results

**Figure 7:**
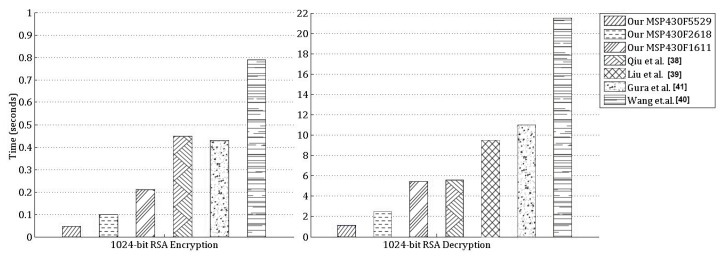
Timings for 1024-bit RSA encryption and decryption.

We use the IAR Embedded Workbench IDE [50] as our development platform, and obtain the timings for the Montgomery multiplication and squaring operations, as well as the RSA encryption and decryption operations, using its cycle counter feature. Our timings for Montgomery multiplication and squaring are given in Table 1. Our timings for 1024-bit RSA encryption and decryption are given in Table 2 and Table 3, and in Figure 7. We compare our timings with the existing 1024-bit RSA implementations in the literature on the same or similar microcontrollers. Our implementation for 1024-bit RSA encryption has the best timings among all, as seen in Table 2 and Figure 7. Note that, similar to our implementation, the works by Qiu et al. [38], Gura et al. [41] and Wang et al. [40] also use the same small exponent $e=2^{16}+1$ for RSA encryption.

In RSA implementations, the decryption operation is more decisive in determining the performance since it is significantly more time consuming than the encryption operation. We give our timings for RSA decryption in Table 3 and Figure 7. We use the CRT to accelerate RSA decryption, similar to Qiu et al. [38], Liu et al. [39], Gura et al. [41] and Wang et al. [40]. We use the sliding window method with the window size of 4 bits, similar to Qiu et al. [38], Liu et al. [39] and Wang et al. [40]. As we use further acceleration techniques, such as recursive subtractive Karatsuba-Ofman, Montgomery multiplication, operand scanning and optimized integer squaring, we achieve better timings as given in Figure 7.

In their work, Qiu et al. give their timings for the 512-bit modular multiplication and squaring operations on MSP430F1611 as 148,777 and 116.252 cycles, respectively [38]. Our 512-bit Montgomery multiplication implementation on the same microcontroller has the timing values of only 35,885 and 32,507 cycles for multiplication and squaring, respectively. Hence, compared to the timings of Qiu et al, our timings are more than 4 and 3 times better for 512-bit integer multiplication and squaring, respectively. According to the theoretical analysis given in Section 3, the cost of RSA decryption using the 4-bit sliding window method and the CRT is roughly 1030 512-bit squarings and 268 512-bit multiplications. Our timings for 512-bit squaring and 512-bit multiplication operations are given in Table 4 as 4.06 ms and 4.48 ms, respectively. Using these figures, the estimated timing for RSA decryption would be 1030×4.06+268×4.48=5382 ms, or 5.38 s which complies with our observed timing value of 5.42 s given in Table 3. The timings of Qiu et al. for 512-bit squaring and 512-bit multiplication operations are also given in Table 4 as 14.53 ms and 18.59 ms, respectively. Using these figures and doing the same analysis, the estimated timing for their RSA decryption implementation would be 1030×14.53+268×18.59=19948 ms, or roughly 20 s, which is nearly 4 times more than their claimed timing value of 5.58 s given in Table 3.

**Figure 8:**
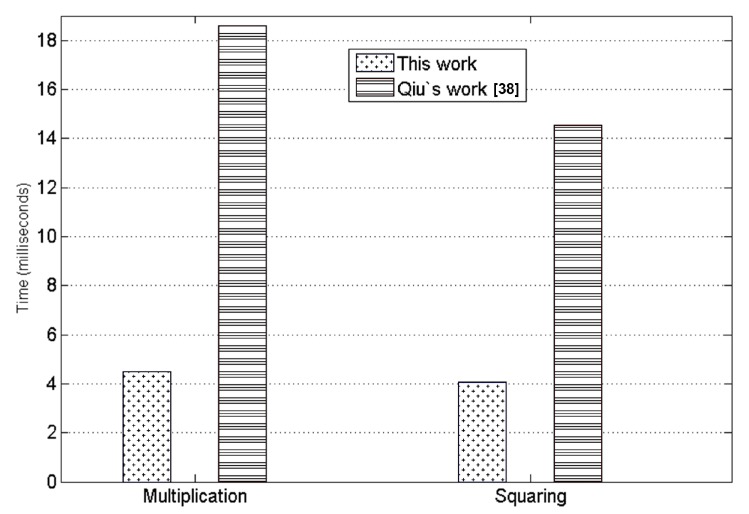
Timings for 512-bit Montgomery multiplication/squaring on MSP430F1611.

Note that Qiu et al. also use the sliding window method with the same 4-bit window size. They do not mention any other acceleration methods they use for their RSA decryption implementation. Hence, it is unclear how they obtained the timing result of 5.58 s for their RSA decryption implementation while the theoretical estimate is 20 s. The comparisons of the timing values for our implementations of the underlying 512-bit modular multiplication/squaring operations and the implementations by Qiu et al. [38], on the same MSP430F1611 microcontroller, are given in Figure 8 and Table 4.

## 5. Conclusions

For solving the key distribution problem in wireless sensor networks, previous works mostly proposed using elliptic curve cryptography, assuming that using RSA would be impractical due to its long key size. With this work, we implemented 1024-bit RSA on the constrained MSP430 microcontroller, a commonly used microcontroller in wireless sensor network nodes. We utilized several methods to accelerate RSA operations and achieved better timings than the existing work in the literature for both the encryption and decryption operations. As future work, we plan to implement RSA on MSP430x, the extended form of MSP430, and improve our timing results by utilizing its extended instruction set. We believe that on MSP430x, RSA with larger key sizes, e.g., 2048-bit or 3072-bit, can also be made feasible with further optimizations.

## Figures and Tables

**Table 1:** 512-bit and 1024-bit Montgomery Multiplication/Squaring Timings.

**512-bit Montgomery Multiplication**		
**Microcontroller**	**# Clock Cycles**	**Time (ms)**
MSP430F5529 @25 MHz	23,272	0.93 ms
MSP430F2618 @16 MHz	33,512	2.09 ms
MSP430F1611 @8 MHz	35,885	4.48 ms
**512-bit Montgomery Squaring**		
**Microcontroller**	**# Clock Cycles**	**Time (ms)**
MSP430F5529 @25 MHz	22,037	0.88 ms
MSP430F2618 @16 MHz	30,437	1.90 ms
MSP430F1611 @8 MHz	32,507	4.06 ms
**1024-bit Montgomery Multiplication**		
**Microcontroller**	**# Clock Cycles**	**Time (ms)**
MSP430F5529 @25 MHz	74,546	2.98 ms
MSP430F2618 @16 MHz	103,975	6.49 ms
MSP430F1611 @8 MHz	112,497	14.06 ms
**1024-bit Montgomery Squaring**		
**Microcontroller**	**# Clock Cycles**	**Time (ms)**
MSP430F5529 @25 MHz	69,504	2.78 ms
MSP430F2618 @16 MHz	95,068	5.94 ms
MSP430F1611 @8 MHz	101,898	12.73 ms

**Table 2:** Timings for 1024-bit RSA encryption.

1024-bit RSA	Microcontroller	# Clock Cycles	Time (s)
Encryption **(This work)**	MSP430F5529 @25 MHz	1,189,089	0.047
Encryption **(This work)**	MSP430F2618 @16 MHz	1,611,482	0.10
Encryption **(This work)**	MSP430F1611 @8 MHz	1,743,445	0.21
Encryption [38]	MSP430F1611 @8 MHz	3,665,144	0.45
Encryption [41]	ATmega128 @8 MHz	-	0.43
Encryption [40]	ATmega128 @8 MHz	-	0.79
Encryption [41]	CC1010 @14 MHz	-	4.48

**Table 3:** Timings for 1024-bit RSA decryption.

1024-bit RSA	Microcontroller	# Clock Cycles	Time (s)
Decryption **(This work)**	MSP430F5529 @25-MHz	28,608,119	1.14
Decryption **(This work)**	MSP430F2618 @16-MHz	40,007,873	2.50
Decryption **(This work)**	MSP430F1611 @8-MHz	43,368,720	5.42
Decryption [38]	MSP430F1611 @8-MHz	44,639,340	5.58
Decryption [39]	ATmega128 @8-MHz	75,680,000	9.46
Decryption [41]	ATmega128 @8-MHz	-	10.99
Decryption [40]	ATmega128 @8-MHz	-	21.5
Decryption [41]	CC1010 @14-MHz	-	106.66

**Table 4:** Timings for 512-bit Montgomery multiplication/squaring.

**Multiplication**	**Microcontroller**	**Clock Cycles**	**Time (ms)**
This work	MSP430F1611 @8 MHz	35,885	4.48 ms
Qiu’s work [38]	MSP430F1611 @8 MHz	148,777	18.59 ms
**Squaring**	**Microcontroller**	**Clock Cycles**	**Time (ms)**
This work	MSP430F1611 @8 MHz	32,507	4.06 ms
Qiu’s work [38]	MSP430F1611 @8 MHz	116,252	14.53 ms
